# Risk and Prevention of Hepatitis B Virus Reactivation during Immunosuppression for Non-Oncological Diseases

**DOI:** 10.3390/jcm10215201

**Published:** 2021-11-08

**Authors:** Lorenzo Onorato, Mariantonietta Pisaturo, Clarissa Camaioni, Pierantonio Grimaldi, Alessio Vinicio Codella, Federica Calò, Nicola Coppola

**Affiliations:** 1 Department of Mental Health and Public Medicine, Faculty of Medicine, University of Campania Luigi Vanvitelli, Via L. Armanni 5, 80138 Naples, Italy; lorenzoonorato@libero.it (L.O.); mariantonietta.pisaturo@unicampania.it (M.P.); clarissacamaioni91@gmail.com (C.C.); peogrimaldi@me.com (P.G.); 2 Infectious Diseases Unit, Azienda Ospedaliera Universitaria Luigi Vanvitelli, Via Pansini 5, 80138 Naples, Italy; alessiovinicio.codella@studenti.unicampania.it (A.V.C.); fede.calo85@gmail.com (F.C.)

**Keywords:** HBV infection, HBV reactivation, rheumatological diseases, gastrointestinal diseases, neurological diseases, dermatological diseases

## Abstract

Reactivation of overt or occult HBV infection (HBVr) is a well-known, potentially life-threatening event which can occur during the course of immunosuppressive treatments. Although it has been described mainly in subjects receiving therapy for oncological or hematological diseases, the increasing use of immunosuppressant agents in non-oncological patients observed in recent years has raised concerns about the risk of reactivation in several other settings. However, few data can be found in the literature on the occurrence of HBVr in these populations, and few clear recommendations on its management have been defined. The present paper was written to provide an overview of the risk of HBV reactivation in non-neoplastic patients treated with immunosuppressive drugs, particularly for rheumatological, gastrointestinal, dermatological and neurological diseases, and for COVID-19 patients receiving immunomodulating agents; and to discuss the potential strategies for prevention and treatment of HBVr in these settings.

## 1. Introduction

Hepatitis B virus (HBV) represents one of the most important threats for public health. According to the WHO estimates, approximately 3.5% of the global population was living with a chronic HBV infection in 2015, and about 900,000 people died during the same year from HBV-related cirrhosis or hepatocellular carcinoma [1]. Furthermore, after HBsAg loss, the viral genome may persist in the hepatocytes, leading to a condition known as “occult HBV infection” (OBI), which is defined as the presence of replication of competent HBV DNA in the liver and/or blood of subjects testing negative for HBsAg [2].

In recent years, the increasing use of immunosuppressive treatments has led to a growing incidence of HBV reactivation (HBVr) in patients with overt or occult infection [3]. As a systematic review recently published by our group estimated a prevalence of OBI in Western countries ranging from 19% to 51% [4], and considering the incidence of immunosuppressive diseases and/or the use of immunosuppressive treatments, the risk of HBVr has become high. Life-threatening reactivation episodes have frequently been described in subjects undergoing immune suppression for oncological or hematological diseases [5,6]; however, little is known about the risk of HBVr in patients treated with immunosuppressants in other settings, such as rheumatological, gastroenterological, neurological, or dermatological diseases, and most recently for SARS-CoV-2 pneumonia.

The present paper was written to provide an overview of the risk of reactivation of HBV infection in non-oncological and non-hematological settings, and to discuss the strategies for preventing and treating these life-threatening events.

## 2. Epidemiology of HBV Infection

HBV belongs to the Hepadnaviridae family, which includes viruses with double-stranded DNA and lipoprotein envelopes. It mainly infects hepatocytes and is one of the most widespread viruses in the world. Despite the incisive vaccination programs that have been carried out, HBV still remains a global health problem due to its enormous burden in terms of morbidity and mortality. In fact, this virus can cause various clinical manifestations: acute hepatitis B, inactive carrier state, and chronic hepatitis B, which can lead to liver cirrhosis and hepatocellular carcinoma [7].

The WHO estimated that in 2015, chronic HBV infections affected about 257 million people in the world, 68% of whom lived in Africa and the Western Pacific [8]. We should point out that the prevalence of infection varies considerably by geographical area: in the African regions, it is 4.6–8.5%; in the Western Pacific region, 6.2–7.6%; in the East Mediterranean regions, the prevalence fluctuates between 2.6% and 4.3%; in South East Asia between 1.5% and 4%; in Europe between 1.2% and 2.6%; and finally, in North America, between 0.7% and 1.6% of the population is estimated to be HBsAg-positive [8].

In the regions with the highest prevalence, infection is mainly transmitted through the vertical route or as a result of intrafamily contacts, and therefore, it is acquired at birth or at an early age. These transmission routes contribute to maintaining high endemicity in these areas, especially given the high rate of chronicization reported when the infection is contracted during the first years of life. In regions with low prevalence, on the other hand, HBV is acquired mainly in adulthood by sexual or parenteral contact; but in these regions high HBsAg prevalence can be found in specific groups, such as among immigrants from areas with high endemicity [9], people who inject drugs (PWID), men who have sex with men (MSM), and people living with HIV (PLWHIV) [10].

Thanks to the extensive vaccination programs carried out, the prevalence of chronic hepatitis B among children under the age of 5 fell to below 1% in 2019 (compared to 5% in the pre-vaccination period or up to 2000), but the goal planned by the WHO—to eliminate HBV infection as a major health problem—is still far from being achieved [11].

## 3. Natural History of HBV Infection

The natural history of chronic HBV infection has been divided into five phases, according to the HBeAg serostatus, the viral load, the transaminase levels, and the grading and staging of liver disease [12]. A HBeAg-positive chronic infection, previously called “immunotolerant phase”, is characterized by a limited or absent immune response against the virus, which leads to intense viral replication, with HBeAg positivity, ALT persistently in the normal range, and no or minimal liver necroinflammation or fibrosis. During the second phase, currently named “HBeAg-positive chronic hepatitis”, the host produces an active immune response against viral antigens, causing a consequent reduction in viral load, and elevation of transaminase levels and liver inflammation. The immune response can eventually lead to control of the infection, with HBeAg seroclearance, low level replication (HBV DNA < 2000 UI/mL), absence of ALT elevation, and mild or no necroinflammation in the liver: these features define the phase of “HBeAg-negative chronic infection”. The acquisition of mutations in the pre-core or basal core promoter regions, however, may allow high-level viral replication despite the presence of antibodies against HBeAg, and lead to elevation of the viral load and liver enzymes, concurrent moderate to severe liver inflammation, and rapid progression of disease. Finally, as previously described, after the HBsAg clearance, the viral genome can still remain detectable in the liver or plasma, defining the “HBsAg-negative phase” or “occult B infection”; several data suggest that the persistence of low-level replication in patients with occult infections can contribute to the advancement of liver fibrosis and the development of hepatocellular cancer in patients with other etiologies of liver disease, particularly hepatitis C virus (HCV) infections [13,14].

In patients with chronic hepatitis, the cumulative 5-year incidence of progression to cirrhosis is estimated to be 8–20%. The wide variability can be determined by the viral load, the HBeAg serostatus, and the presence of concomitant alcohol abuse or coinfection with HCV, hepatitis Delta virus (HDV), or human immunodeficiency virus (HIV) [15]. Once cirrhosis occurs, the risk of decompensation is estimated to be around 20–25% per year, and the 5-year survival is 20–30% [16]. Moreover, about 5–15% of cirrhotic patients develop hepatocellular cancer during their lives [17].

## 4. HBV Reactivation following Immunosuppressive Treatments

Although many definitions have been proposed, HBV reactivation commonly refers to either the de novo detection of HBV DNA or a ≥10-fold increase in HBV DNA level compared with the baseline value in HBsAg-positive patients, and seroconversion to HBsAg-positive status in previously negative subjects [18]. The biological basis of HBVr is the persistence of the viral genome as ccc-DNA in liver cells.

Several risks factors for the occurrence of reactivation after immune suppression have been identified [19].

As regards the host characteristics, the most important factor is the HBV immune control preceding the treatment. Obviously, patients with chronic HBV infections are significantly more at risk of reactivation compared to subjects with OBI, as demonstrated by several studies in both oncological [20] and non-oncological settings [21,22]. Furthermore, among HBsAg-negative subjects, the presence of anti-HBs has been related to a lower risk of reactivation, as demonstrated by Seto et al. [23] among 63 patients receiving rituximab for hematological malignancies. Other host factors that have been related to increased risk of reactivation include male gender [24], older age [25], and underlying lymphoproliferative diseases [5].

According to the agent used and the serostatus of the patient, the risk of HBVr can be roughly classified into high risk (frequency of reactivation > 10% in the absence of prophylaxis), medium risk (1–10%), or low risk (<1%) [18,26,27]. It is well known that the use of specific drugs, such as B-cell depleting agents, is associated with a high risk of reactivation in patients with both overt and occult infections [28]. Other drug classes that can cause reactivation in more than 10% of cases among HBsAg-positive subjects include anthracycline derivatives [20] and high dose corticosteroids (20 mg daily of prednisone or equivalent) administered for more than 4 weeks of treatment [18]. In particular, glucocorticoids have been demonstrated to directly stimulate HBV replication in hepatoma cells through the activation of regulatory elements [29]. Additional drug classes that have been related to reactivation in patients with overt and/or occult HBV infections include inhibitors of TNF-alfa [22] or other cytokine or integrin inhibitors [30], and tyrosine kinases [31] and JAK inhibitors [21]. Many of these agents have been used increasingly in recent years in non-oncological settings, such as rheumatological diseases or inflammatory bowel diseases, but less clear data on their impacts on HBV infection are available so far among non-oncological populations.

## 5. Strategies for the Prevention and Treatment of HBV Reactivation

**Figure 1:**
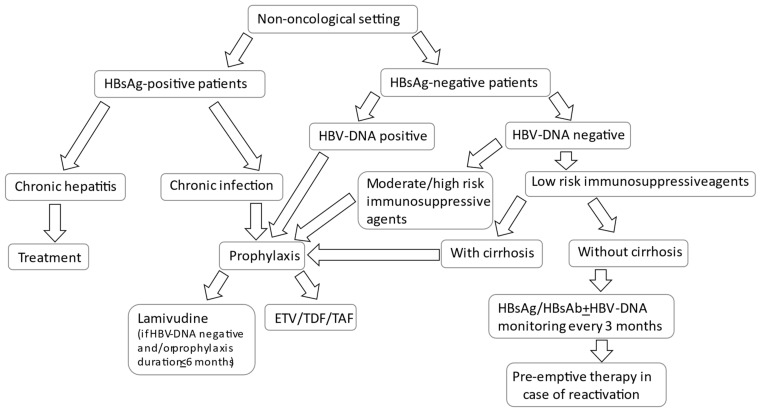
Algorithm of HBVr management in patients undergoing immunosuppression for non-oncological diseases.

Since the likelihood of HBV reactivation depends on the type, duration, and intensity of the immunosuppression, therapeutic strategies aimed at avoiding HBV reactivation are modulated according to the risk profile of reactivation. The algorithm of HBVr prevention according to HBsAg serostatus and risk of reactivation is shown in Figure 1.

Considering the phases of HBV infection, the patients with chronic hepatitis are at high risk for HBVr and progression of liver damage, so and they should be evaluated for treatment. Those with HBeAg-positive or HBeAg–negative chronic infections and those with previous infections show various risks of reactivation, and should be evaluated for HBV prophylaxis or pre-emptive therapy according to the immunosuppressive regimens they will undergo [12].

In the patients with HBV-related hepatitis, a therapy with high genetic barrier nucleos(t)ide analogues (entecavir (ETV), tenofovir (TDF), or tenofovir alafenamide (TAF)) should be started as soon as possible, as for non-immunocompromised subjects, so this is considered therapy and not prophylaxis [12,32,33,34,35].

Instead, in patients with HBV infections undergoing treatments that involve moderate–high risks of reactivation, some form of prophylaxis should be prescribed, preferably an antiviral drug with a high genetic barrier, i.e., ETV, TDF, or TAF, as recommended by international guidelines [12,32]. Lamivudine was the first nucleoside analogue used as antiviral prophylaxis to reduce HBVr complications; however, the development of resistance in patients requiring prolonged duration of therapy may lead to a re-emergence of HBV DNA and a risk of HBVr, so newer nucleoside agents with high barriers to resistance may provide additional options for antiviral prophylaxis [36]. According to the guidelines of the American Gastroenterological Association (AGA), the use of lamivudine should be limited to the prophylaxis of patients with undetectable viral loads at baseline or with expected durations of prophylaxis of less than 6 months [32].

The prophylaxis should be started before the prescription of the immunosuppressive regimen and continued until 12–18 months after the discontinuation of the treatment [32,37,38,39].

HBsAg-negative, anti-HBc-positive patients should be considered for antiviral prophylaxis or pre-emptive therapy according to the HBV DNA at baseline.

Patients with positive HBV DNA at baseline should receive prophylaxis similarly to patients with overt infections. Conversely, the management depends on the risk of HBV reactivation: when the risk of reactivation is high (e.g., the use of B-cell depleting agents), a prophylactic agent should be prescribed. In the case of a moderate risk of reactivation, the correct strategy is still a matter of debate. The guidelines of the European Association for Study of Liver (EASL) recommend a pre-empitve therapy strategy [12], although a prophylaxis can be considered, according to the AGA guidelines [32]. Instead, with a low risk of reactivation, a strategy based on pre-emptive therapy is generally recommended. Patients should undergo close monitoring during and after the immunosuppression. If HBsAg seroreversion occurs, antiviral therapy with a nucleos(t)ide analogue should be initiated.

However, in select patients with previous infection and low risk of reactivation, but with advanced liver disease due to a different etiology, lamivudine prophylaxis may be considered, to avoid the risk of a life-threatening hepatic failure following the reactivation [12,32,40,41].

As regards the monitoring of the patients for whom prophylaxis is envisaged, liver function tests and HBV DNA for patients with HBV infections and HBV DNA/HBsAg tests for patients with resolved HBV infection should be performed every 3–6 months during prophylaxis and up to 12–18 months after stopping the antiviral treatment, as an episode of reactivation can still occur after the interruption of the antiviral therapy. Instead, in patients with HBV related diseases for whom therapy is envisaged, virological and biochemical monitoring is lifelong [12,32].

In the following sections we discuss the risk of reactivation related to the use of immunosuppressant agents in non-oncological settings and provide an overview of the possible management strategies.

### 5.1. Risk of HBV Reactivation in Gastroenterological Diseases

Autoimmune and inflammatory disorders, such as Crohn’s disease and ulcerative colitis, are common gastroenterological conditions that often require the use of immunosuppressive therapies. Disease severity and the relapsing and remitting course affect the selection of the right drug. Agents commonly used include corticosteroids, immunomodulator agents (e.g., azathioprine/mercaptopurine and methotrexate), biological therapies (i.e., tumor necrosis factor (TNF) inhibitors), anti-adhesion therapy, anti-IL12/23 p40 antibody, and Janus kinase (JAK) inhibitor in the ultra-refractory cases.

A list of studies evaluating the risk of HBV reactivation in patients with gastroenteric diseases treated with immunosuppressive agents is reported in Table 1. In a retrospective study of 8887 patients treated with TNF inhibitors for autoimmune disorders, HBVr was observed in 9 of the 23 HBsAg-positive patients and 2 of 4267 patients in the unknown HBV status group [22]. Concomitant immunosuppressives, including steroids and non-biological immunosuppressants, were also associated with HBVr.

In a Spanish multicenter analysis of 162 patients with inflammatory bowel disease (IBD) treated with different immunosuppressant drugs, the authors described HBVr in 9 (36%) out of the 25 HBsAg-positive patients; however, none of the HBsAg-negative but anti-HBc positive subjects patients reported HBVr [42]. In addition, the authors found that treatment with ≥2 immunosuppressive agents was an independent predictor of HBVr (OR 8.75; 95% CI 1.16–65.66).

Morisco et al. [43], in a retrospective study, evaluated 5096 patients with IBD and found a lower rate of HBVr; indeed, HBVr was detected in only one of six (16%) HBsAg-positive patients treated with a therapeutic regimen that included infliximab and azathioprine.

In a systematic review evaluating 257 subjects with positive hepatitis B markers (89 were HBsAg-positive and 168 were HBsAg-negative/anti-HBc-positive carriers) treated with anti-TNF inhibitors for IBD and other autoimmune disorders, Perez-Alvarez et al. [44] found a lower rate of reactivation in patients who had antiviral prophylaxis (23% vs. 62%, *p* = 0.003) but a higher rate in those already treated with immunosuppressive drugs (96% vs. 70%, *p* = 0.033).

In a national cohort of 3357 patients with IBD in the USA, Shah et al. did not identify a single case of confirmed clinically relevant HBVr after anti-TNF starting [45].

As regards HBsAg-negative/anti-HBc-positive subjects with IBD, only a few cases of HBVr have been described. Clarke et al. [46] investigated the prevalence of HBVr in a single-center retrospective cohort analysis of 120 HBsAg-negative/anti-HBc-positive subjects (anti-TNF treatment in 19% of the cases), and found a low rate of HBV reactivation (0.8%). Solay et al. [47] assessed 29 cases of patients with resolved HBV infection who received biological treatment: HBVr was observed in five patients (17.2). Pauly et al. reported that not one of the 178 HBsAg-negative/anti-HBc-positive subjects treated with TNF antagonists had documented HBVr [22]. Additionally, Papa et al. [48] reported no cases of HBV reactivation in 22 HBsAg-negative/anti-HBc-positive IBD patients treated with anti-TNF. Additionally, no HBVr and/or associated biochemical breakthrough was detected in a retrospective study that evaluated 90 patients (of whom 13 with IBD) with past HBV infection who received anti-TNF treatment [49]. In the above-mentioned systematic review [44], the authors reported an HBVr rate of 39% in HBsAg-positive patients and 5% in HBsAg-negative/anti-HBc-positive patients.

In conclusion, complete serology for HBV is required in IBD patients to determine the virological status (active carrier, inactive carrier or anti-HBc positivity), since the HBV profile affects the choice of HBV therapy, prophylaxis, or monitoring. In fact, in consideration of the higher risk of reactivation, IBD patients who are HBsAg-positive carriers should receive prophylactic antiviral treatment with nucleotide or nucleoside analogues before the introduction steroids at moderate to high doses (>20 mg/die of prednisone or equivalent) for more than 4 weeks, azathioprine, anti-TNF therapy, or ustekinumab. Treatment may be lifelong in patients with chronic HBV and for at least one year after discontinuing immunosuppressive therapy in HBsAg asymptomatic carriers.

The approach to IBD patients who are HBsAg-negative and anti-HBc-positive is not standardized across the various guidelines. The American Gastroenterological Association recommends antiviral prophylaxis for HBsAg-negative/anti-HBc-positive patients treated with anti-TNF or with corticosteroids (10–20 mg or >20 mg prednisone daily for 4 weeks); there is a moderate risk of reactivation in this population [32]. The European Association for the Study of the Liver [12] and the European Crohn and Colitis Organization guidelines [50] suggest the strategy of active monitoring of viremia and recommend that antiviral agents be initiated once HBV DNA or seroconversion to positive HBsAg is detected. Therefore, considering the indications of the published guidelines and the scant data available in the literature in support of antiviral prophylaxis, HBsAg monitoring every 2–3 months may be recommended for such patients.

### 5.2. Risk of HBV Reactivation in Dermatological Diseases

Immunosuppressive drugs, both conventional and biological, are used in many different dermatological diseases, among which psoriasis is the most common and affects approximately 125 million people worldwide [51]. Conventional disease modifying drugs (cDMARDs) include acitretine, cyclosporin A, and methotrexate; and biological DMARDs (bDMARDs) include etanercept, adalimumab, infliximab, ustekinumab, golimumab, certolizumab, and secukinumab. Trials investigating new drugs do not usually involve HBV patients, so data on their safety regarding HBV reactivation in patients with psoriasis are based mostly on case reports and small retrospective cohort studies.

Table 2 summarizes the studies evaluating reactivation of current and past HBV infections in the setting of dermatological disease.

Chiu et al. [52] evaluated the risk of reactivation of HBV in 14 psoriatic patients undergoing therapy with ustekinumab. Two out of the seven (29%) HBsAg-positive patients not receiving prophylaxis showed HBVr during ustekinumab treatment, whereas no reactivation was observed among the 3 HBsAg-negative/HBcAb-positive patients.

In a retrospective cohort study, Ting et al. [53] included 54 subjects with active or previous infections. Only three patients experienced virological reactivation. The calculated incidence rate of annual HBV reactivation with ustekinumab was 17.4% among inactive HBV carriers without prophylaxis and 1.5% in the occult hepatitis B infection group.

A retrospective study by Snast et al. [54] reported no reactivation among 25 psoriatic patients with past infections and one with a current HBV infection treated with biological therapies. Similar results were observed in an Italian study among patients with psoriasis and chronic HBV infection treated with adalimumab [55].

In a cohort study published in 2018 [56], the authors followed-up 32 patients with psoriasis and concurrent positive HBV markers (chronic inactive and occult cases) treated with biological agents (adalimumab, etanercept, ustekinumab) for at least 24 weeks and found no evidence of viral reactivation 3 months after stopping treatment.

Finally, a meta-analysis performed by Cantini et al. [57] estimated a pooled prevalence of HBVr of 3% among subjects with previous HBV infections, and of 15.4% in patients with overt infections during treatment with anti TNF for dermatological and rheumatological diseases.

Although the reliability of many of these studies is limited by small numbers of subjects and short periods of follow-up, the available evidence suggests that in HBsAg-positive patients who receive treatment with immunosuppressive drugs associated with moderate risk of HBV reactivation (anti-TNFα, including etanercept, adalimumab, and golimumab; and cytokine or integrin inhibitors, such as ustekinumab or secukinumab), antiviral prophylaxis would be preferable; for HBsAg-negative/anti-HBc–positive patients, both antiviral prophylaxis and close monitoring with pre-emptive therapy are feasible options.

### 5.3. Risk of Reactivation in Rheumatological Diseases

Rheumatological drugs include corticosteroids, non-steroidal anti-inflammatory drugs (NSAIDs), analgesic drugs, and disease-modifying antirheumatic drugs (DMARDs). The latter are divided into conventional synthetics (cs) and biological (b) drugs.

The csDMARDs include sulfasalazine; methotrexate; hydroxychloroquine; leflunomide; and less frequently, azathioprine, gold salts, and minocycline [58]. The bDMARDs can instead be distinguished on the basis of mechanism of action into TNF inhibitors (etanercept, infliximab, adalimumab, certolizumab, and golimumab), IL-1 inhibitors (anakinra, and canakinumab), IL-6 and IL-6R inhibitors (tocilizumab and sarilumab, respectively), inhibitors of IL-17 (secukinumab and ixekizumab), IL-23 inhibitors (ustekinumab and guselkumab), and JAK kinase inhibitors (tofacitinib, baricitinib, upadacitinib, filgotinib, and peficitinib [59,60].

Studies that provided data on HBVr in patients with rheumatological diseases are shown in Table 3. HBVr has been found in rheumatological patients receiving both csDMARD and bDMARD treatment. Among the csDMARDs, methotrexate has been widely used, but its impact on HBVr has not yet been clarified. A Thai study did not report episodes of reactivation among 65 HBcAb-positive, HbsAg-negative rheumatological patients treated for nine years with methotrexate [61], whereas seven cases of reactivation, five of which were severe, were described in the literature among patients with overt HBV infections treated with the same drug [62,63,64,65,66].

A study by Chen et al. [67] enrolling 123 HbsAg-positive subjects with rheumatoid arthritis from 2006 to 2012 demonstrated a higher risk of HBVr (occurring in 30 patients) when csDMARDS were combined with other immunosuppressants: in particular, low-dose glucocorticoids with csDMARDs and bDMARDs (excluding rituximab) caused an HBV reactivation in 54.5% of cases, bDMARDs (excluding rituximab) associated with csDMARDs coincided with reactivation in 5.9% of patients, and the risk of HBVr for csDMARDs associated with glucocorticoids was 12.5%. Regarding outcomes, despite antiviral treatment being initiated at the time of HBVr appearance, 13 (43.3%) patients developed severe hepatitis and 5 (16.7%) hepatic decompensation, with death in three cases.

As regards anti-TNF bDMARDs, a study by Ryu et al. [68] recorded two (6.9%) cases of HBVr within one year of anti-TNF treatment among 29 patients not receiving a primary prophylaxis. Only one reactivation (9%) was recorded in the prophylaxis group (20 patients) at the 64th week of therapy with bDMARDs.

Regarding HbsAg-negative patients, a prospective multicenter study by Fukuda et al. [69] described an HBVr rate of 1.93/100 people/year among 1042 patients undergoing immunosuppressive treatment for rheumatological diseases. The incidence of reactivation among patients testing negative for HBsAb was significantly higher than that of HBsAb-positive subjects (4.32 vs. 1.42/100 persons/year). No liver dysfunction occurred during HBVr. In a subsequent paper on the same group, 57 cases of HBVr (0.43/100 persons/year) occurred over the course of 4 years; age >70 years [70]. The presence of isolated anti-HBc antibodies and immunosuppressive therapy other than monotherapy with methotrexate were found to be independent risk factors for HBVr. The authors proposed a scoring system to distinguish between patients at higher and lower risks of reactivation. Finally, in a meta-analysis published in 2013, Lee et al. [71] reported eight (1.7%) cases of HBV reactivation among 468 patients with previous HBV infections who underwent anti-TNF treatment for rheumatic disease.

In a study by Varisco et al. [72], HBVr was evaluated in patients with previous HBV infections treated with methotrexate plus rituximab (with or without steroids) for rheumatoid arthritis. None of the subjects received antiviral prophylaxis for HBV. No case of seroreversion to HbsAg positivity was observed, but 6 out of 28 (21%) HBsAb-positive patients presented a decrease (>50%) in the antibody titer. Only one patient was positive for HBV DNA after 6 months of treatment with rituximab; the subject was promptly treated with lamivudine, avoiding an exacerbation of hepatitis.

In a study by Urata et al. [73], 135 HBsAg-negative and anti-HBc-positive patients undergoing immunosuppressive treatment for rheumatoid arthritis were prospectively evaluated. HBV DNA was positive during follow-up in seven cases (5.1%). The patients who received bDMARDs had a significantly higher rate of HBV reactivation compared with those who were treated with other immunosuppressants (*p* = 0.008), with a hazard ratio of 10.9 (1.4–87.7).

Nakamura et al. [74] evaluated 57 patients with rheumatoid arthritis treated with bDMARDs and a previous HBV infection, with a median observation of 18 months. No antiviral prophylaxis was prescribed. HBV DNA became positive in 5.3% of the population, specifically in two patients treated with tocilizumab and in one patient treated with etanercept. However, there were no significant changes in markers of liver function, and no patient required antiviral therapy.

Regarding the JAK kinase inhibitors, a study by Harigai et al. [75] included 215 patients with previous or current HBV infections, treated with baricitinib (with or without csDMARDs) for rheumatoid arthritis, in four clinical trials. All patients tested negative for HBV DNA at baseline. During the follow-up, 32 (14.9%) patients tested positive for HBV DNA, but only four of them met the HBV reactivation criteria (HBV DNA ≥ 100 IU/mL). The use of baricitinib was discontinued temporarily in two patients and permanently in four. In no case was there clinical evidence of hepatitis.

In conclusion, the risk of HBVr depends on the subject’s immunosuppressive status and the baseline condition of HBV infection. For both HBsAg-positive and HBsAg-negative patients, the risk of HBVr is low with csDMARDs, including methotrexate, leflunomide, and azathioprine; and short (<4 weeks), low-dose (<10 mg/day of prednisone or equivalent) cortisone-based therapies. However, it is moderate with anti-TNFs (etanercept, infliximab, and adalimumab) or tyrosine kinase inhibitors (such as baricitinib), and is even higher with combination therapies; thus, an antiviral prophylaxis should be recommended in these cases [18,76].

### 5.4. Risk of HBV Reactivation in Neurological Diseases

Multiple sclerosis (MS) is a chronic inflammatory disease of the central nervous system (CNS) causing demyelination, that causes progressive neurodegeneration and disability. Glucocorticoids have been widely used to manage the acute phases of the disease, although their effectiveness tends to decrease over time. However, disease modifying drugs acquired a central role in the treatment of MS outpatients. So far, a series of biological therapies have been approved which are distributed in the first, second, and third lines of treatment. All have some degree of immunosuppressive potential: they mainly include anti-CD20 monoclonal antibodies (ocrelizumab, ofatumumab), anti-CD52 antibodies (alemtuzumab), a4b1 integrin inhibitor (Natalizumab), DNA intercalator (Mitoxantrone), and sphingosine-1 phosphate inhibitor (fingolimod) and its modulators (siponimod, ozanimod) [77].

Although all these drugs seem to be related to HBVr, limited data are available in the literature concerning the risk of HBVr when used in a neurological setting. Ciardi et al. [78] reported a case of HBVr in a patient with a previous HBV infection treated with ocrelizumab for multiple sclerosis who did not receive prophylaxis. Although many authors agree on the need for antiviral prophylaxis in patients treated with ocrelizumab, similarly to what is recommended for other anti-CD20 antibodies, no clear and definitive consensus exists on the best prevention strategies in subjects receiving other immunosuppressive drugs in this setting [79]. Further studies are needed to clarify this point.

### 5.5. Risk of HBV Reactivation in COVID-19 Patients

The SARS-CoV-2 pandemic has been responsible for more than 150 million cases and over 3 million deaths as of April 2021, according to the data reported on the online dashboard implemented by the Johns Hopkins University [80]. Several immunosuppressive and immunomodulating agents have been adopted for the treatment of COVID-19, such as corticosteroids, which are currently recommended by the WHO guidelines for severe or critical diseases [81], and interleukin-6 (IL-6) inhibitors, which have been tested in several clinical trials compared to the standard of care [82].

As already stated, high dose glucocorticoids significantly increase the risk of reactivation in both overt and occult B infections [18]. However, limited data are available in the literature on the HBVr rate of COVID-19 patients treated with corticosteroids. A retrospective study reported three cases of HBVr, two of whom had received methylprednisolone treatment, among 20 Chinese HBsAg-positive, treatment-naïve patients hospitalized for SARS-CoV-2 pneumonia between January and March 2020 [83].

Regarding the IL-6 inhibitors, several studies have reported an increased risk of HBVr in patients receiving tocilizumab for rheumatological diseases [84]. However, a retrospective cohort study [85] including 29 HBsAg-negative/anti-HBc-positive patients receiving immunosuppressive treatment for COVID-19 (mostly tocilizumab or siltuximab) reported no case of HBVr requiring antiviral treatment. Only two patients showed detectable HBV DNA during the follow-up, in both cases below the limit of quantification (<15 UI/mL). Most probably, the short duration of treatment limits the risk of reactivation in this setting. No data are available on the risks related to other immunosuppressive agents that have been used in COVID-19 patients, such as baricitinib, ruxolitinib, and tofacitinib; however, these drugs have been associated with significant risks of HBVr reactivation in other settings [75]. Therefore, all patients expected to be treated with corticosteroids for more than 7–10 days or with other immunosuppressive drugs for COVID-19 pneumonia should be screened for current and previous HBV infections, in order to evaluate the risk of reactivation and implement preventive strategies when needed.

## 6. Conclusions

The growing use of immunosuppressive treatments in patients with a large variety of non-oncological diseases has drawn attention to the risk of reactivation of HBV infection in many settings. However, the available data on the risk of HBVr related to the different drugs are limited and fragmentary. Further prospective studies are needed to assess the best preventive strategies to reduce the occurrence of these potentially life-threatening events.

## Figures and Tables

**Table 1:** Studies on HBV reactivation in patients with gastroenteric diseases.

First Author, Year [Reference]	Study Design	N. Patients	Gastroenteric Disease (*n*, %)	Immunosuppressive Treatment	HBV Status (*n*, %)	HBVr Definition	HBVr (*n*, %)	HBVr among Pts Receiving Prophylaxis (*n*, %)	HBVr among Pts Not Receiving Prophylaxis (*n*, %)
Pauly MP, 2018 [22]	Retrospective	8887	1186 (13.4)	Anti-TNF α agents ± steroids and non-biological immunosuppressants	HBsAg+: 23 (0.3) HBsAg−/HBcAb+: 178 (2)	>1 log increase in serum HBV DNA from baseline OR serum HBV DNA detectable when previously undetectable OR HBV DNA >2000 IU/mL if no baseline OR reverse seroconversion from HBsAg negative to HBsAg+.	HBsAg+: 9 (39.1) HBsAg−/HBcAb+: 0 (0)	HBsAg+: 1 (11.1) HBsAg−/HBcAb+: 0 (0)	HBsAg+: 8 (88.9) HBsAg−/HBcAb+: 0 (0)
Loras C, 2010 [42]	Retrospective	162	162 (100)	Steroids ± anti-TNF α agents ± non-biological immunosuppressants	HBsAg+: 25 (15.4) HBsAg−/HBcAb+: 65 (40.1)	1.5–2 fold the baseline value of alanine transaminase (ALT) plus an increase of >2000 IU/ml HBV DNA levels or DNA reappearance in a negative patient.	HBsAg+: 9 (36) HBsAg−/HBcAb+: 0 (0)	HBsAg+: 1 (11.1) HBsAg−/HBcAb+: 0(0)	HBsAg+: 8 (88.9) HBsAg−/HBcAb+: 0(0)
Morisco F, 2013 [43]	Retrospective	5096	5096 (100)	Anti-TNF α agents ± non-biological immunosuppressants	HBsAg+: 6 (0.1) HBsAg−/HBcAb+: 4 (0.07)	In HBsAg−positive patients >1 log10 increase HBV DNA with or without the concomitant increase in transaminases; in isolated anti-HBc-positive, the re-emergence of HBsAg or appearance or at least >1 log10 increase HBV DNA	HBsAg+: 1 (16.6) HBsAg−/HBcAb+: 1 (25)	HBsAg+: 0(0) HBsAg−/HBcAb+: 0(0)	HBsAg+: 1 (100) HBsAg−/HBcAb+: 0 (0)
Perez-Alvarez, 2011 [44]	Retrospective	257	20 *	Anti-TNF α agents	HBsAg+: 89 (34.6) HBsAg−/HBcAb+: 168 (65.3)	The reappearance of serum HBV-DNA in a patient with previously inactive or resolved HBV infection, and either as an increase of >1 log10 of viral load or >400 IU/mL (2000 copies/mL) with respect to the baseline HBV-DNA load before anti-TNF therapy, or as the appearance of serum HBV DNA above standard cutoff values (>60 IU/L, equivalent to >300 copies/mL)	HBsAg+: 35 (39.3) HBsAg−/HBcAb+: 9 (5.3)	HBsAg+: 7 (20) HBsAg−/HBcAb+: 0 (0)	HBsAg+: 28 (80) ** HBsAg−/HBcAb+: 0 (0)
Shah R, 2018 [45]	Retrospective	3357	3357 (100)	Anti-TNF α agents	HBsAg+: NA HBsAg−/HBcAb+: NA	Using ICD-9 codes for HBV (070.2×, 070.3×), acute liver failure (670××), or filled prescriptions for medications used in the treatment of HBV. Potential cases of HBV reactivation were verified using manual EMR through CAPRI.	HBsAg+: 0 (0) HBsAg−/HBcAb+: 0 (0)	HBsAg+: 0 (0) HBsAg−/HBcAb+: 0 (0)	HBsAg+: 0 (0) HBsAg−/HBcAb+: 0 (0)
Clarke WT, 2018 [46]	Retrospective	3171	23 ***	Anti-TNF α agents ± immunomodulators	HBsAg+: NA HBsAg−/HBcAb+: 120 (3.8)	Demonstration of reverse seroconversion to positive HBsAg status OR development of detectable HBV DNA	HBsAg+: NA HBsAg−/HBcAb+: 1 (0.8)	HBsAg+: NA HBsAg−/HBcAb+: NA	HBsAg+: NA HBsAg−/HBcAb+:NA
Solay AH, 2018 [47]	Retrospective	278	1 ****	Anti-TNF α agents	HBsAg+: NA HBsAg−/HBcAb+: 29	Detection of HBV DNA and/or HBsAg conversion in blood analysis during the follow-up.	HBsAg+: NA HBsAg−/HBcAb+: 5 (17.2)	HBsAg+: NA HBsAg−/HBcAb+: 0 (0)	HBsAg+: NA HBsAg−/HBcAb+: 0 (0)
Papa A, 2013 [48]	Prospective	301	301 (100)	Anti-TNF α agents	HBsAg+: 1 (0.3) HBsAg−/HBcAb+: 22 (7.3)	HBsAg or HBV-DNA detection in patients previously negative for HBsAg or with undetectable levels of HBV DNA	HBsAg+: 0 (0) HBsAg−/HBcAb+: 0 (0)	HBsAg+: 0 (0) HBsAg−/HBcAb+: NA	HBsAg+: 0 (0) HBsAg−/HBcAb+: NA
Sayar S, 2020 [49]	Retrospective	653	13 *****	Anti-TNF α agents ± corticosteroid and/or immunomodulator.	HBsAg+: 5 (0.8) HBsAg−/HBcAb+: 90 (13.8)	An increase of >1 log10 IU/mL in the HBV DNA level compared with the past value OR positivity in those who were HBV DNA negative OR detection of any positive HBV DNA level in patients whose baseline HBV DNA level was not studied	HBsAg+: NA HBsAg−/HBcAb+: 0 (0)	HBsAg+: NA HBsAg−/HBcAb+: 0 (0)	HBsAg+: NA HBsAg−/HBcAb+: 0 (0)

NA: not available; * number of IBD patients out of 44 patients with HBVr; ** in two patients, whether antiviral prophylaxis was done was not specified; *** number of IBD patients out of the 120 HbsAg−/HBcAb+ patients; **** number of IBD patients out of the 29 HbsAg−/HBcAb+ patients; ***** number of IBD patients out of the 90 HbsAg−/HBcAb+ patients.

**Table 2:** Studies on HBV reactivation in patients with dermatological diseases.

First Author, Year [Reference]	Study Design	N. Patients	Dermatological Disease	Immunosuppressive Treatment	HBV Status (*n*, %)	HBVr Definition	HBVr (*n*, %)	HBVr among Pts Receiving Prophylaxis (*n*, %)	HBVr among Pts Not Receiving Prophylaxis (*n*, %)
Chiu, 2013 [52]	Retrospective cohort	14	Psoriasis	Ustekinumab	HBsAg+: 11 HBsAg−/HBcAb+: 3	One of the following: 1. ALT elevation with increase in serum HBV DNA level to >1 log10 copies/ mL higher than before therapy; 2. Absolute increase in HBV DNA level exceeding 6 log10 copies /mL; 3. Conversion of serum HBV-DNA test results from negative to positive.	HBsAg+: 2 (14.3) HBsAg−/HBcAb+: 0 (0)	HBsAg+: 0 (0) HBsAg−/HBcAb+:0 (0)	HBsAg+: 2 (28.6) HBsAg−/HBcAb+:0 (0)
Ting, 2018 [53]	Prospective cohort	54	Psoriasis	Ustekinumab	HBsAg+: 10 HBsAg−/HBcAb+: 44	One of the following: 1. >2 log increase in HBV replication from baseline levels, 2. New appearance of HBV DNA at a level of >100 IU/mL in a person with previously stable or undetectable levels 3. Detection of HBV DNA at a level >20,000 IU/mL in a person with no baseline HBV DNA	HBsAg+: 2 (20) HBsAg−/HBcAb+: 1 (2.3)	HBsAg+: 0 (0) HBsAg−/HBcAb+:0 (0)	HBsAg+: 2 (25) HBsAg−/HBcAb+:1 (2.3)
Snast, 2017 [54]	Retrospective cohort	26	Psoriasis	Adalimumab, Etanercept, Golimumab, Infliximab, Secukinumab, Ustekinumab	HBsAg+: 1 HBsAg−/HBcAb+: 26	One of the following: 1. Increase in HBV replication of at least 1 log10 copies/mL 2. Conversion of serum HBV DNA results from negative to positive	HBsAg+: 0 (0) HBsAg−/HBcAb+: 0 (0)	HBsAg+: 0 (0) HBsAg−/HBcAb+: 0 (0)	HBsAg+: 0 (0) HBsAg−/HBcAb+: 0 (0)
Piaserico, 2017 [55]	Retrospective cohort	17	Psoriasis	Adalimumab	HBsAg+: 10 HBsAg−/HBcAb+: 7	One of the following: 1. ALT elevation with increase in serum HBV DNA level to >1 log10 copies/ mL higher than before therapy; 2. Absolute increase in HBV DNA level exceeding 6 log10 copies /mL; 3. Conversion of serum HBV-DNA test results from negative to positive.	HBsAg+: 0 (0) HBsAg−/HBcAb+: 0 (0)	HBsAg+: 0 (0)	HBsAg+: 0 (0) HBsAg−/HBcAb+: 0 (0)
AlMutairi, 2018 [56]	Prospective cohort	32	Psoriasis	Adalimumab, Etanercept, Ustekinumab	HBsAg+: 4 HBsAg−/HBcAb+: 28	One of the following: 1. Increase in serum HBV DNA level to >1 log10 copies/mL higher than before therapy; 2. Absolute increase in HBV DNA level exceeding 6 log10 copies /mL; 3. Conversion of serum HBV-DNA test results from negative to positive.	HBsAg+: 0 (0) HBsAg−/HBcAb+: 0 (0)	HBsAg−/HBcAb+: 0 (0)	HBsAg+: 0 (0)

**Table 3:** Studies on HBV reactivation in patients with rheumatological diseases.

First Author, Year [Reference]	Study Design	N. Patients	Rheumatological Disease	Immunosuppressive Treatment	HBV Status (*n*, %)	HBVr Definition	HBVr (*n*, %)	HBVr among Pts Receiving Prophylaxis (*n*, %)	HBVr among Pts Not Receiving Prophylaxis (*n*, %)
Laohapand, 2015 [61]	Cross-sectional	173	RA, spondyloarthropathies, systemic lupus erythematosus, others	Methotrexate	HBsAg+: 1 (0.6) HBsAg−/HBcAb+: 65 (37.6)	Not reported	HBsAg+: 0 (0) HBsAg−/HBcAb+: 0 (0)		HBsAg+: 0 (0) HBsAg−/HBcAb+: (0)
Chen, 2017 [67]	Retrospective cohort	123	RA	Glucocorticoids: 51 (41.5) bDMARDS: 36 (29.3) csDMARDs: 123 (100)	HBsAg+: 123 (100)	One of the following: 1. Increase in HBV DNA >1 Log10 IU/mL compared with baseline 2. 3-fold increase in serum alanine aminotransferase (ALT) level accompanied by HBV DNA >20,000 copies/mL	HBsAg+: 30 (24.4)		HBsAg+: 30 (24.4)
Ryu, 2012 [68]	Retrospective cohort	49	RA, ankylosing spondylitis	Etanercept, Infliximab, adalimumab	HBsAg+: 49 (100)	Both the following: 1. 10-fold rise in HBV DNA compared with baseline resulting in HBV DNA greater than 20,000 IU/mL (HBeAg-positive patients) or 2000 IU/mL (HBeAg-negative patients), 2. Increase in AST or ALT to above twice the upper normal limit (40 IU/l)	HBsAg+: 3 (6.1)	HBsAg+: 1 (5.0)	HBsAg+: 2 (6.9)
Fukuda, 2019 [70]	Prospective cohort	1127	RA, others	Glucocorticoids: 373 (38.9) bDMARDS: 274 (28.8) csDMARDs: 751 (79.1)	HBsAg−/HBcAb+: 1127 (100)	Positive conversion of HBV-DNA	HBsAg−/HBcAb+: 57 (5.1)		HBsAg−/HBcAb+: 57 (5.1)
Varisco, 2016 [72]	Retrospective cohort	33	RA	Rituximab ± DMSARDs	HBsAg−/HBcAb+: 33 (100)	HBsAg seroreversion or serum HBV-DNA positivity	HBsAg−/HBcAb+: 0 (0)		HBsAg−/HBcAb+: 0 (0)
Urata, 2011 [73]	Prospective cohort	123	RA	Glucocorticoids, bDMARDS, csDMARDs	HBsAg−/HBcAb+: 123 (100)	Not reported	HBsAg−/HBcAb+: 7 (5.2)		HBsAg−/HBcAb+: 7 (5.2)
Nakamura, 2016 [74]	Retrospective cohort	57	RA	bDMARDs	HBsAg−/HBcAb+: 57 (100)	Serum HBV-DNA positivity	HBsAg−/HBcAb+: 3 (5.3)		HBsAg−/HBcAb+: 3 (5.3)
Harigai 2020 [75]	Prospective cohort	290	RA	Baricitinib, Methotrexate	HBsAg−/HBcAb+: 215 (74.1) HBsAg−/HBcAb−: 75 (25.9)	≥2 log increase from baseline levels or new appearance of HBV DNA to a level of ≥100 IU/mL	HBsAg−/HBcAb+: 32 (14.8) HBsAg−/HBcAb−: 4 (5.3)		HBsAg−/HBcAb+: 32 (14.8) HBsAg−/HBcAb−: 4 (5.3)

Footnotes: RA: rheumatoid arthritis.
